# A Prospective Cohort Study on the Safety of Infant Pentavalent (DTwP-HBV-Hib) and Oral Polio Vaccines in Two South Indian Districts

**DOI:** 10.1097/INF.0000000000002594

**Published:** 2020-04-14

**Authors:** Narendra Kumar Arora, Manoja Kumar Das, Ramesh Poluru, Neeraj Kumar Kashyap, Thomas Mathew, John Mathai, Mahesh Kumar Aggarwal, Pradeep Haldar, Thomas Verstraeten, Patrick L. F. Zuber, the INCLEN Vaccine Safety Study Group

**Affiliations:** From the * The INCLEN Trust International, New Delhi, India; † Department of Community Medicine, Government Medical College, Thiruvananthapuram, Kerala, India; ‡ Department of Pediatrics, PSG Institute of Medical Sciences and Research, Coimbatore, Tamil Nadu, India; § Ministry of Health and Family Welfare, Government of India, New Delhi, India; ¶ P95, Epidemiology and Pharmacovigilance Consulting and Services, Leuven, Belgium; ‖ Department of Essential Medicines and Health Products, World Health Organization, Geneva, Switzerland; the ** INCLEN Vaccine Safety Study Group members are listed in the Appendix.

**Keywords:** pentavalent vaccine, oral polio vaccine, serious adverse events, hospitalization, death

## Abstract

**Background:**

Safety of pentavalent (DTwP-HBV-Hib) vaccine has been a public concern in India and other countries. This study attempted to document the association of serious adverse events following immunization (AEFI, including hospitalizations and deaths of all causes) with the 3 doses of pentavalent and oral poliovirus (OPV) vaccines.

**Methods:**

A cohort of 30,688 infants in 2 south Indian districts were enrolled and followed-up between October 2014 and May 2016, following their first vaccination with DTwP-HBV-Hib and OPV at public health facilities. During weekly follow-ups, by telephone or home visits, the serious AEFIs (hospitalizations and deaths) occurring any time after each vaccination until 4 weeks after third dose were documented. The incidence risk ratios (IRRs) of serious AEFIs in the first (days 0–6) and fourth weeks (days 21–27) after the vaccine doses were compared using the poisson regression analysis.

**Results:**

Of the 30,688 infants enrolled, 30,208 received their third doses of vaccines. During the 4-week periods following each vaccination, there were 365 hospitalizations and 17 deaths. Adjusted incidence risk ratio of 3 doses combined for post-vaccination serious AEFIs during the first week compared with fourth week was 0.8 [95% confidence interval: 0.6–1.0].

**Conclusions:**

There was no increased risk of a serious AEFIs during the first week after any of the 3 doses of pentavalent and OPV vaccination compared with the fourth week. In the absence of any temporal clustering, mortality and hospitalization rates observed in vaccinated infants probably reflects the natural occurrence of such events.

## Abstract 2

Supplemental Digital Content is available in the text.

Vaccination has been one of the major success stories in global health, leading to the eradication of deadly diseases like smallpox and possibly the virtual disappearance of some diseases from several regions.^1^ Over the last 25 years, effective vaccination programs have resulted in a >90% decrease in the global disease burdens for diphtheria, pertussis, and tetanus, including India.^2–4^ However, in spite of the extensive evidence of their benefits, concerns and controversies about the value and safety of vaccines continue to impact adversely their acceptance in diverse contexts. Public concerns over reports linking autism with measles, mumps, and rubella vaccine in the United Kingdom had negative impact on vaccine coverage causing several disease outbreaks,^5^ which spread to other countries, such as the United States, France, Italy, and the Netherlands.^6–9^ This misinformation has spread globally. The rumor mongering through social media considerably affected vaccine coverage during the MR campaigns in south India.^10^ The growing global challenge of vaccine hesitancy relates largely to vaccine safety concerns, leading to public confidence erosion, which in turn dampens the vaccine coverage.^11^ The Global Advisory Committee on Vaccine Safety emphasized the need for thorough investigation and causality assessment of serious adverse events following immunization (AEFI) as a quality assurance mechanism for immunization programs.^12^

The decreased disease burdens in the developed countries have largely been due to the successful implementation of immunization programs using multivalent combination vaccines. The vaccines against diphtheria, tetanus, and pertussis combined (DTwP) are in use for several decades under universal immunization program. Recently the HBV and *Haemophilus influenza* type b (Hib) have been added to DTwP, known as the pentavalent (DTwP-HBV-Hib) vaccine. Over the past 15 years Global Alliance for Vaccines and Immunizations has supported the introduction of pentavalent vaccine in developing countries for protection against invasive Hib disease in universal immunization program.^13^ However, there were temporary interruptions and introduction delays in Sri Lanka, Bhutan, Vietnam^14^ and Pakistan^15^ during 2011–2016 due to reports of hospitalizations and deaths following vaccination after national launch of the pentavalent vaccine. Temporal association of vaccinations and sudden infant death syndrome (SIDS) has been the subject of extensive speculation, despite several studies having refuted any causal association,^16–18^ particularly after the diphtheria-pertussis-tetanus immunization.^19^

In India, the reports of 32 infant deaths following the pentavalent vaccination as part of the national AEFI surveillance between 2011 and 2013 raised concerns, although no causal association could be identified.^20^ Some professionals and civil society groups, however, appealed before the Supreme Court of India to ban the pentavalent vaccine.^21,22^ Although the safety of pentavalent vaccine has been documented in the pre-licensure studies,^23,24^ these studies were too small to identify rare adverse events. Thus, it was deemed necessary to carry out further investigations on this issue. This study was designed in consultation with all key stakeholders including the Ministry of Health and Family Welfare, Government of India and WHO (advised by Global Advisory Committee on Vaccine Safety), to assess the risk of serious AEFIs (hospitalizations and deaths) following pentavalent vaccination in an infant cohort from 2 Indian states, where pentavalent vaccine was first introduced.

## MATERIALS AND METHODS

The pentavalent vaccine (manufactured by Serum Institute of India Ltd., Pune, India) was first introduced in the states of Kerala and Tamil Nadu in December 2011 with a 6, 10 and 14 weeks schedule for 3 primary doses. Both states have efficient health systems with high vaccination coverage^25^ and low infant-mortality rates (IMR): 12 and 21 per 1000 live births in 2013 in Kerala and Tamil Nadu, respectively, compared with an overall IMR of 40 across India.^26^ This study was conducted in 2 districts, Kollam (Kerala State) and Coimbatore (Tamil Nadu State).

### Study Design

This prospective self-controlled cohort study enrolled infants who received their first dose of pentavalent and oral poliovirus (OPV) vaccine from the public health system between October 2014 and November 2015 in Kollam district, and between May 2015 and February 2016 in Coimbatore district.

### Recruitment and Data Collection

Infants who received their first dose of pentavalent and OPV vaccines at all public health facilities in the 2 districts (District, Special and Taluk Hospitals, Community Health Centers, Primary Health Centers, Sub-centers) and out-reach points of rural and urban areas were traced based on the contact details recorded in the immunization registers. Home visits were made to confirm eligibility (received first dose of vaccine, expected to stay in the district for the 6 months follow-up period and contactable by telephone/mobile) and to enroll infants after obtaining informed written consent. Data on maternal care, infant gender, date of birth, birth weight, pregnancy-related and neonatal illnesses were collected at enrollment. The parent(s) were contacted weekly by telephone or home visit to document the health of the infant including any illness, hospitalization or death. The follow-up contact was continued even if the second and third vaccine doses were delayed due to any reason. Study staff confirmed receipt of the second and third vaccine doses from the vaccination card and the health facility register. If infants received their second and third vaccine doses at private facilities, a copy of the vaccination card was collected as proof of date of exposure. For each dose, the vaccine batch number, date and place of administration was documented. Recruited infants were followed up to 4 weeks after the third dose of the vaccines or death, whichever was earlier. Data were collected on tablets using a customized program developed with open source platforms (Android, PHP and MySQL version 5.1) and data including other materials uploaded, synchronized in the server and stored/deposited in a central data repository. An independent International Advisory Group (IAG) with vaccine safety experts and Brighton Collaboration representation supervised the study implementation including field visits. The IAG reanalyzed the data for validation.

### Outcomes

This study captured the serious AEFIs which included both hospitalizations and deaths due to any cause during the follow-up period. The details about the events such as clinical features, diagnosis, treatment, period and place of hospitalization were captured. For hospitalizations, the study team visited the hospital for confirmation and followed till outcome to document the information. Hospitalization episodes of at least 24-hours as inpatient were further categorized as “appropriate,” “inappropriate” and “indeterminate” using the Pediatric Appropriateness Evaluation Protocol–India criteria. The PAEP-India tool for child and newborn were adapted from the available tools and validated in hospitalized patients from multiple hospitals in India.^27^ Each case was assessed by 2 independent pediatricians using the PAEP-India tool for child and categorized the case based on checklist criteria. A third pediatrician was consulted in cases of mismatch between the 2 reviewers. For deaths, the study confirmed the event by home visit, followed by verbal autopsy by senior investigator 4 weeks later. Causes of death were determined independently by 3 pediatricians trained in review of verbal autopsies and also referred to available hospital records.

### Data Analysis

The 4 weeks following every dose of vaccination were considered to be the observation period with weekly intervals post-vaccination as separate risk periods (Figure, Supplemental Digital Content 1, http://links.lww.com/INF/D758). As almost all the serious AEFI (deaths and hospitalizations) reported in the passive national AEFI surveillance program occurred during the first week after any vaccine dose, the first week was considered as the high-risk period and the fourth week as the comparison period. It was hypothesized that, the risks of serious AEFI due to any cause, during the first and fourth weeks post-vaccination remain similar for each dose of the vaccines.

Person-time of observation was calculated based on the time from vaccine administration and the date of occurrence of an event, the date of administration of the next vaccine dose (if <28 days), the last contact date (if lost to follow-up), date of death or the first 28 days after vaccination when the next dose was delayed, according to the recommended schedule, whichever came first. Follow-up was defined by weekly risk windows after each vaccine dose split into first (0–6 days), second (7–13 days), third (14–20 days) and fourth (21–27 days) weeks. If a subsequent dose was given before the end of the fourth week, the follow-up time was censored and follow-up period was assigned to the subsequent dose. If the next dose was delayed, the person-time beyond 27 days after each dose were excluded from the analysis. Poisson regression analysis was used to estimate the incidence rates (IRs) and incidence rate ratios (IRRs) for serious AEFIs (hospitalization and all-cause deaths) during the 4-consecutive weekly risk periods after each dose separately and for all 3-doses combined. IRs were calculated by dividing the number of events by the corresponding person-time denominator and IRRs were calculated by dividing the IR in the risk period of interest by the IR in the fourth week. In a multivariate model, the IRR was adjusted for age at start of risk period (continuous, in days) and seasonality (quarterly; January–March, April–June, July–September, and October–December). To assess the potential of healthy vaccinee bias, we performed a sensitivity analysis to compare the risks in the first week after vaccination following the second and third doses with the risks in the fourth weeks of the preceding doses. All statistical analyses were performed using Stata version 15.0 (StataCorp LLC, TX). In addition, all deaths documented were reviewed by the national AEFI committee for causality assessment according to WHO published methodology.^20^

### Sample Size

Considering the IMR in the study states to be 12–21 per 1000 live births and a neonatal mortality rate (0–28 days) of 6–15 per 1000 live births,^26^ the post-neonatal mortality (29–365 days) was expected to be 6 per 1000 live births. This translated to ~0.125 infant deaths per 1000 live births per week. As there was no information about the hospitalization:death ratio in this age group from India, we assumed that for every infant death there would be ~4 hospitalizations. The required sample size was 27,422 infants to detect an IRR for hospitalizations and or deaths of 1.8 in the analyses pooling the different doses, assuming a 5% significance level (α = 0.05) and 80% power (β = 0.2).^28^ With the available sample (30,688), the observed results show that between the risk periods, we are able to detect a risk-ratio of 2.5 and 1.5 (at a 5% significance level and 80% power) of serious AEFIs (hospitalization and deaths), for individual doses and all 3-doses combined, respectively.

### Ethical Approvals

The study protocol and related documents were approved by ethics committees of participating institutes. The infants were recruited after obtaining informed written consent from parents or legally authorized representatives. The study protocol and related documents were approved by Institute Ethics committees at The INCLEN Trust International (INCLEN) (vide IIEC-021 dated October 14, 2013), Government Medical College, Thiruvananthapuram (vide 01/01/2014 dated January 17, 2014), PSG Institute of Medical Sciences and Research, Coimbatore (vide 13/351 dated March 5, 2014) and World Health organization, Geneva (vide RPC625 dated January 22, 2014).

## RESULTS

**FIGURE 1.**
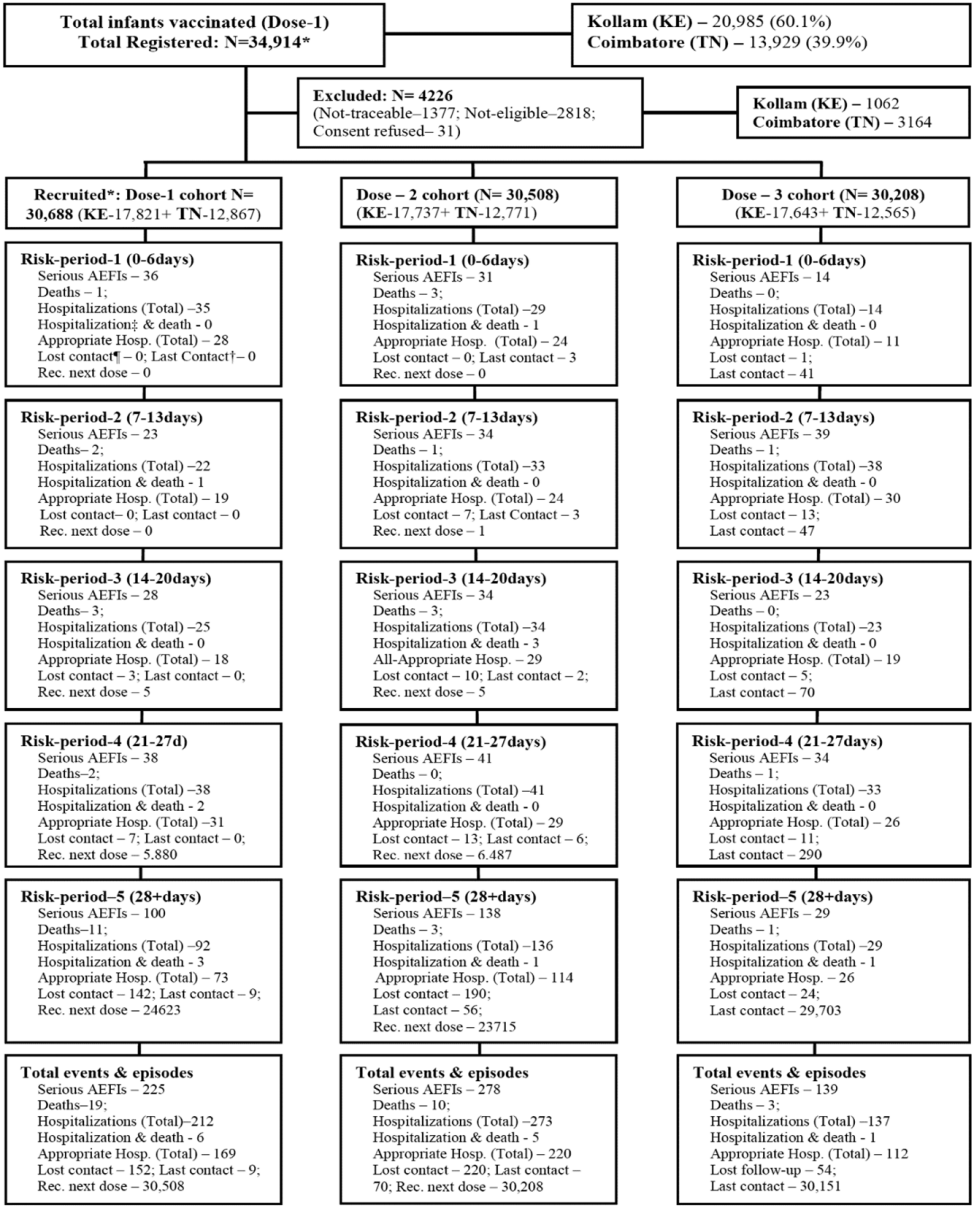
Selection and follow-up of the study population in the study districts and distribution of events. *Received first dose at public health facilities; ¶ lost contact is “Lost contact during the period”; †last contact defined as “Received respective vaccination and time period at last follow-up contact”; ‡appropriate hospitalizations as per expert opinion and identified using PAEP criteria. KE-Kerala; TN-Tamil Nadu.

During the study period, 34,914 infants received their first dose of pentavalent and OPV vaccines at the public health facilities. Of these, 1377 (3.9%) infants could not be traced, 2818 (8.1%) were not eligible (1287 were not staying in the district for 6 months, and 1531 were from other districts) and consent was refused for 31 (0.1%) infants. Of the 30,688 infants recruited, 30,208 (98.4%) received their third vaccine dose and 29,728 (96.9%) completed the scheduled follow-up (Fig. 1). Of 479,523 scheduled weekly follow-up contacts (including the contacts during the intervals between the different vaccine doses till completion of 4 weeks after third dose), 89.9% were completed with averages of 4.4 (dose 1), 4.8 (dose 2) and 4.9 (dose 3) contacts for the vaccine doses (data not shown).

Baseline characteristics of the cohort are summarized in Table 1. No age or sex differences were observed across the districts. The Kollam population had a higher proportion of low and median socio-economic status and joint or extended families. Median ages of infants were 48, 83 and 118 days at the times of the first, second and third vaccine doses, respectively. A total of 622 hospitalizations and 32 deaths were documented during the follow-up period.

**TABLE 1.**
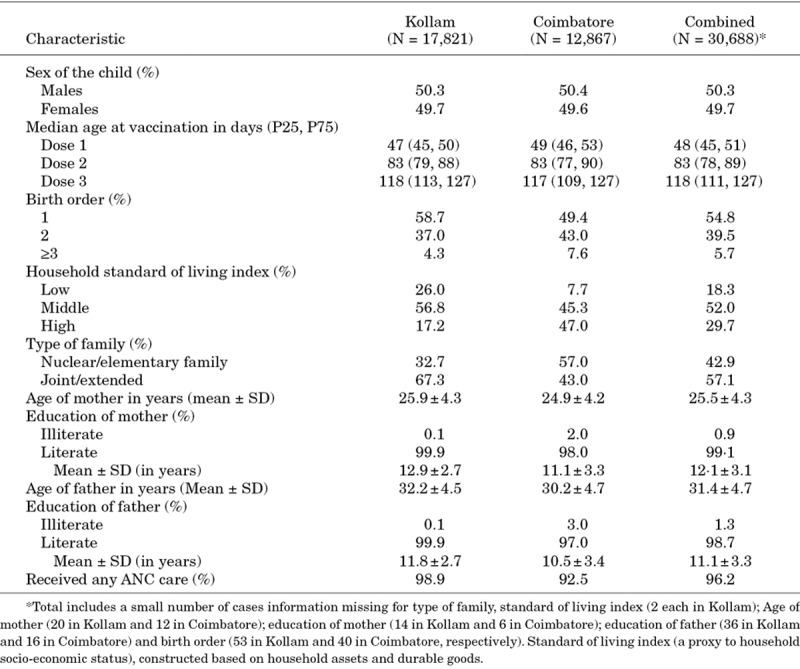
Characteristics of Study Infants and Their Households

Within the 4 weeks after the 3 vaccine doses (per protocol study risk periods), a total of 365 hospitalizations (120, 137 and 108 after doses 1, 2 and 3, respectively) were documented. Of these 365 hospitalizations, 288 were classified as appropriate (96, 106 and 86 after doses 1, 2 and 3, respectively). A total of 257 hospitalizations (92, 136 and 29 after doses 1, 2 and 3, respectively) occurred after fourth weeks of the first and second doses of vaccination (Fig. 1). Pneumonia/acute respiratory infection (70.1%) was the leading cause followed by diarrhea (6.4%), fever (4.3%) and urinary tract diseases (3.1%) during the 4 week and post-4 week periods after the vaccine doses (Table, Supplemental Digital Content 2, http://links.lww.com/INF/D759).

**FIGURE 2.**
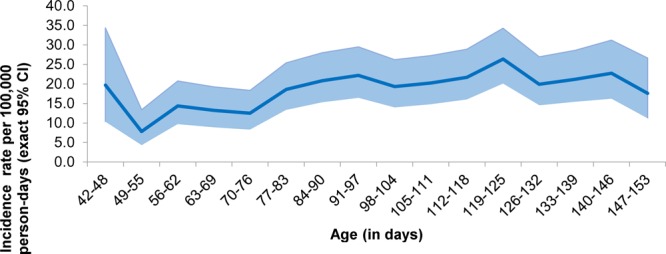
Serious AEFI (hospitalization and all-cause deaths) incidence rates (per 100,000 person-days with exact 95% CI as shaded area) by age (independent of vaccination windows). Incidence per 100,000 person-days (with exact 95% CI as shaded area); Week defined as 6-days period, starting on day-0; Person-time starts from age at first dose of vaccination and duration until the first and last day of the age period or until the Serious AEFI (hospitalization/death) events (succeeding vaccinations not considered). The hospitalization incidence presented in the figure is between 42 and 153 days of age, where 98.6% [(3,402,860/3,451,027)*100] of follow-up calls/visits were made.

**FIGURE 3.**
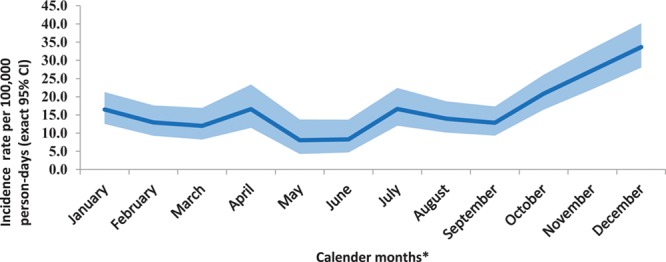
Serious AEFIs (hospitalization and all-cause deaths) incidence rates (per 100,000 person-days with exact 95% CI) by calendar months. Incidence per 100,000 person-days (with exact 95% CI); *Calendar month with 30-days period, irrespective of study year period; Person-time starts from the first day of the month and until the last day of the month period or until the Serious AEFI events (hospitalization/death).

Of the 32 total deaths observed, 17 deaths (8,7 and 2 after doses 1, 2 and 3, respectively) occurred within 4 weeks after 3 vaccine doses, while 15 deaths (11, 3 and 1 after doses 1, 2 and 3, respectively) occurred during post-4 weeks period, (Fig. 1). The leading causes of death included infections (31.3%), congenital malformations (28.1%), congenital heart diseases with infections (15.6%), SIDS (15.6%) and other causes (9.4%) (Table, Supplemental Digital Content 2, http://links.lww.com/INF/D759). Almost all congenital malformations were diagnosed prior to death. The SIDS was made based on Brighton Collaboration Criteria.^29^ Causality assessment by the National AEFI committee classified all deaths as coincidental. Differentials in causes of hospitalization and death during the first 4-weeks (0–27 days) and post-4 weeks (28+ days) after vaccination were small (Table, Supplemental Digital Content 2, http://links.lww.com/INF/D759). Figures 2 and 3 show the IRs of serious AEFIs (hospitalization and deaths) by age and seasonality. Higher serious AEFI (hospitalization and deaths) rates were observed during 91 and 146 days of age (Fig. 2); between October and December, with a peak in December due to higher prevalence of respiratory illnesses (Fig. 3).

### Serious AEFIs (Hospitalization and Deaths) Within 4 Risk-periods

The occurrence of serious AEFIs (hospitalization and deaths) across the risk-periods for the 3 doses are summarized in Table 2. Within the 4 weeks after the 3 vaccine doses, a total of 375 serious AEFIs (365 hospitalizations, 17 deaths and 7 hospitalization and deaths) occurred (125, 140 and 110 after doses 1, 2 and 3, respectively) (Fig. 1). For all-3 doses combined, the adjusted IRRs of serious AEFIs within the first week compared with the fourth week were 0.8 [95% confidence interval, (CI): 0.6–1.0]. Compared with the fourth week, the adjusted IRRs of serious AEFIs for the first week after first, second and third doses were 1.2 (95% CI: 0.8–1.9), 0.9 (95% CI: 0.6–1.5) and 0.5 (95% CI: 0.3–0.9), respectively. IRRs for serious AEFIs were not significantly higher in any of the other risk periods (second or third weeks) either for the 3 doses separately or for all doses pooled. Analyses of hospitalizations according to appropriateness did not show any increased risk in any week compared with the fourth week after any dose (Tables, Supplemental Digital Content 3, http://links.lww.com/INF/D760, and Supplemental Digital Content 4, http://links.lww.com/INF/D761). The sensitivity analysis comparing the rates of serious AEFI in the first week after the second or third doses with the fourth week after the first or second doses did not show any differences, suggesting the absence of a healthy vaccinee effect (Table 2).

**TABLE 2.**
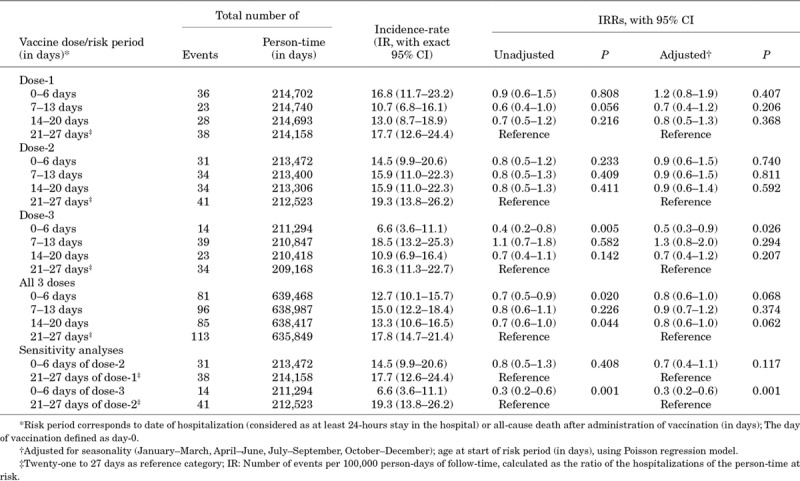
Serious AEFIs (Hospitalization and All-Cause Deaths) IRs and IRRs (Unadjusted and Adjusted) After Pentavalent and Oral Polio Vaccines: Combined for Both Districts, Kollam (Kerala) and Coimbatore (Tamil Nadu), India

## DISCUSSION

This is the first large study to examine the association between administration of routine infant pentavalent and OPV vaccines and serious AEFIs (all-cause hospitalizations and/or deaths) in India, using a self-controlled prospective cohort design. We have shown that the unadjusted and adjusted risks of hospitalizations and deaths after any of the 3 scheduled doses of routine vaccines were similar during the first and fourth weeks post-vaccination, for either each dose separately, or all doses combined. Due to low death rates, we could not perform analyses for each individual dose but the combined analysis and analyzing the first dose only did not indicate any death clusters following vaccination.

In a highly immunized population, self-controlled study designs with defined risk windows for outcome assessment are best suited to assess possible effects during a specific time period as they are not susceptible to control selection.^30^ Activation of the immune response to different vaccines occurs at different time points according to the nature of the antigen.^18,31–34^ Hence, in the current study, we considered the first week after vaccination to represent the period of highest risk and the fourth week to be the comparison period. The findings of our study refute the concern of an association between serious adverse events and the pentavalent vaccine.

The main strength of the study is the representativeness of the study cohort with respect to the population in the study area due to the high rates of participation and completion of follow-up. The socioeconomic status of the population enrolled in the study was highly representative of the overall population in the 2 districts. Use of electronic data collection enabled timely follow-up and accurate data capture with real-time monitoring. Documentation of hospitalization from both participants and hospitals allowed confirmation of causes of hospitalizations and deaths. In addition, the availability of source documents and vaccination records ensured accurate confirmation of vaccine exposure.

The main limitation was possible selection bias, as infants received first vaccine dose in the private system were not represented in the study. However, according to recent National Family Health Survey (NFHS-4, 2015–2016) data, 78% and 86% of children 12–23 months of age in Kerala and Tamil Nadu, received their vaccines from a public health facility.^25^ We could not trace 3.9% infants due to inaccurate address information and 8.1% could not be included due to moving out or residing outside of the 2 districts. To control for confounding by contraindication to vaccination, pre-vaccination time intervals were not included in the control periods in the main analysis. Sensitivity analyses performed to assess the potential of a healthy vaccinee bias did not suggest presence of this. The study was also limited in detecting small changes for deaths, despite the large sample size.

The study found that 128 serious adverse events (120 hospitalizations and 8 deaths) occurred among 30,000 infants during the 4 weeks after receiving their first dose of pentavalent vaccine. Calculating based on Black et al^35^ and projected on a 26 million annual Indian birth cohort, it is estimated that with national coverage of 100% at a minimum 26,500 infants per year will suffer a serious health event within 1 week of receiving their first dose of pentavalent vaccine, or there will be at least 21,200 events with the current 3-dose coverage of ~80% for pentavalent vaccine.^25^ These events reflect prevailing morbidities and mortality that would have occurred regardless of vaccination as documented in 2 Indian districts with low infant mortality. Investigating these events to ensure that the vaccine product or its administration are safe remains a fundamental function of the vaccine pharmacovigilance system. This highlights the importance of continued monitoring of common serious health problems in the infant population to measure the health burden and provide new epidemiologic information on the true causes of morbidity and mortality in this group.

The study did not find any increased risk of serious AEFIs (all-cause hospitalizations or deaths) in the first week following pentavalent (and OPV) vaccination and the occurrence of these AEFIs were just coincidental. That is, in the absence of temporal clustering, mortality and hospitalization rates observed in vaccinated children reflect the natural occurrence of such events in that age range in this part of India. The study findings should help to address the vaccine safety concerns and controversies around the pentavalent vaccine in India and other low- and middle-income countries, and so boost public confidence in the program. Suspicion about a possible relationship between the vaccination and serious AEFIs among parents, communities and the health providers may not only threaten the success of immunization programs but also potentially hinder introduction of newer vaccines.

## CONCLUSIONS

Close monitoring of over 30,000 infants during and after receiving their first 3 doses of DTwP-HBV-Hib and OPV did not identify any relationship between the vaccinations and the occurrence of serious AEFIs (hospitalization and/or death). The study did document the huge number of serious coincidental events that can be expected even when vaccines are properly administered. In view of the recent inclusion of several new vaccines into the immunization program in India, the findings of this study will be very useful in addressing vaccine hesitancy and safety concerns to increase public acceptance and coverage.

## ACKNOWLEDGEMENTS

We express our gratitude to the district health officials and health functionaries at the public health facilities in the 2 districts for their constant support. We are highly appreciative of the parents and families of all infants for consenting to participate in this study and for their cooperation during follow-up. We thank the GAVI, the Vaccine Alliance through World Health Organization (WHO) and the patronage from Ministry of Health and Family Welfare, Government of India, New Delhi, India and the Departments of Health and Family Welfare, Government of Kerala and Tamil Nadu States for funding to conduct the study. We also acknowledge the guidance and continued support provided by the technical experts and representatives from WHO, Technical Advisory Group (TAG) members, International Advisory Group (IAG) members, officials from the Ministry of Health and Family Welfare at National and State levels. We appreciate the support from Tom Cattaert and Germano Ferreira, P95 Pharmacovigilance and Epidemiology, Heverlee, Belgium, for the validation of results, and Keith Veitch (keithveitch communication) for editorial guidance.

## Supplementary Material



## APPENDIX

*INCLEN Vaccine Safety Study Group: Rajamohanan K. Pillai, MD**, Sivamani Manivasagan, MD††, Sairu Philip, MD‡‡, Geetha S, MD**, Ramesh S, MD‡, Anil Mathew, PhD††, Ravindra Mohan Pandey, PhD§§, Sanjay Chaturvedi, MD‖, Jyoti Joshi, MD¶¶, Siddarth Ramji, MD***, Harish Chellani, MD†††, Rajib Dasgupta, PhD‡‡‡, Satinder Aneja, MD§§§, D. K. Taneja, MD‖‖, K. Kolandaswamy, DPH¶¶¶, P. K. Jameela, MD****, J. Radhakrishnan, MVSc¶¶¶, K. Ellangovan, MD****, R. Sadanandan, MPhil****, Ajay Khera, MD§, Rakesh Kumar, MD††††, Sunil Bahl, MD‡‡‡‡, Sujit Jain, MD‡‡‡‡, Madhur Gupta, MD‡‡‡‡, Jan Bonhoeffer, MD§§§§, Miriam C. J. M. Sturkenboom, PhD‖‖, and Christine G. Maure, MD¶*.

‡Department of Pediatrics, PSG Institute of Medical Sciences and Research, Coimbatore, Tamil Nadu, India; §Ministry of Health and Family Welfare, Government of India, New Delhi, India; ¶Department of Essential Medicines and Health Products, World Health Organization, Geneva, Switzerland; **Department of Pediatrics, Government Medical College, Thiruvananthapuram, Kerala, India; ††Department of Community Medicine, PSG Institute of Medical Sciences and Research, Coimbatore, Tamil Nadu, India; ‡‡Department of Community Medicine, T.D. Medical College, Alappuzha, Kerala, India; §§Department of Biostatistics, All India Institute of Medical Sciences, New Delhi, India; ‖‖Department of Community Medicine, University College of Medical Sciences, Delhi, India; ¶¶Center for Disease Dynamics, Economics & Policy, New Delhi, India; ***Department of Paediatrics, Maulana Azad Medical College, New Delhi, India; †††Department of Paediatrics, Vardhman Mahavir Medical College and Safdarjung Hospital, New Delhi, India; ‡‡‡Department of Social Medicine and Community Health, Jawaharlal Nehru University, New Delhi, India; §§§Department of Paediatrics, School of Medical Sciences & Research, Sharda University, Greater Noida, Uttar Pradesh, India; ‖‖‖Department of Community Medicine, Maulana Azad Medical College, New Delhi, India; ¶¶¶Department of Health and Family Welfare, Tamil Nadu, India; ****Department of Health and Family Welfare, Kerala, India; ††††United Nations Development Programme, New Delhi, India; ‡‡‡‡World Health Organization, New Delhi, India; §§§§University of Basel Children's Hospital, Basel & Brighton Collaboration, Switzerland; and ‖‖‖‖Julius global Health University Medical Center Utrecht, The Netherlands.
