# Supplementary material for: A Prospective Cohort Study on the Safety of Infant Pentavalent (DTwP-HBV-Hib) and Oral Polio Vaccines in Two South Indian Districts
Source: Pediatr Infect Dis J. 2020 Apr 14;39(5):389–96. doi: 10.1097/INF.0000000000002594 (PMC7170438; doi:10.1097/INF.0000000000002594)
Supplement: Supplementary file 1 [file inf-39-389-s001.docx]

**SDC 1.** Description of the observation periods and risk periods for comparison.


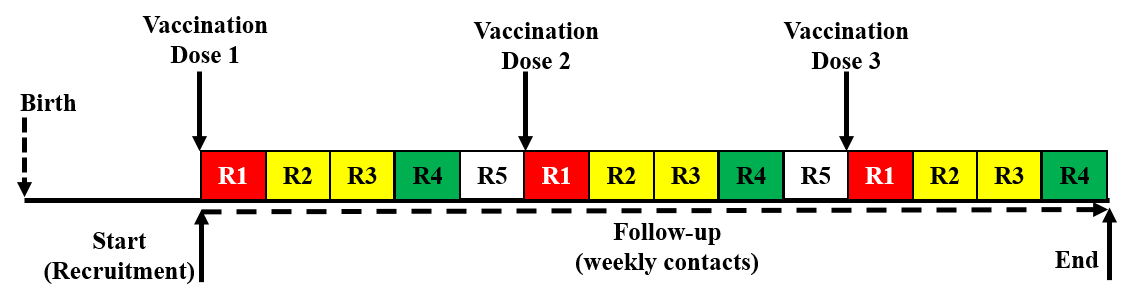


***Risk periods:*** **R1:** *First week* (0-6 days, high risk); **R2:** *Second week* (7-13 days, intermediary risk); **R3:** *Third week* (14-20 days, intermediary risk); **R4:** *Fourth week* (21-27 days, low risk); **R5:** ≥28 days to till next dose or end of follow-up or lost contact (variable, range from days to weeks)
