# Supplementary material for: A Prospective Cohort Study on the Safety of Infant Pentavalent (DTwP-HBV-Hib) and Oral Polio Vaccines in Two South Indian Districts
Source: Pediatr Infect Dis J. 2020 Apr 14;39(5):389–96. doi: 10.1097/INF.0000000000002594 (PMC7170438; doi:10.1097/INF.0000000000002594)
Supplement: Supplementary file 2 [file inf-39-389-s002.docx]

**Supplemental Digital Content 2.** Cause of Hospitalization and deaths (within four weeks and after four weeks) after pentavalent and oral polio vaccines: Combined for both districts, Kollam (Kerala) and Coimbatore (Tamil Nadu), India.

| Categories | Within four weeks of any vaccine dose (0-27 days) | After four weeks of any vaccine dose (28+ days) | Total N (%) |
| --- | --- | --- | --- |
| **Hospitalization diagnosis** |  |  |  |
| Pneumonia/ARI | 249 (68.2) | 187 (72.8) | 436 (70.1) |
| Diarrohea | 24 (6.6) | 16 (6.2) | 40 (6.4) |
| Fever | 18 (4.9) | 9 (3.5) | 27 (4.3) |
| Cong. Malformation | 11 (3.0) | 9 (3.5) | 20 (3.2) |
| UTI | 13 (3.6) | 6 (2.3) | 19 (3.1) |
| CNS Disease | 13 (3.6) | 2 (0.8) | 15 (2.4) |
| Sepsis | 10 (2.7) | 4 (1.6) | 14 (2.3) |
| Febrile seizure | 9 (2.5) | 1 (0.4) | 10 (1.6) |
| Intussusception | 1 (0.3) | 3 (1.2) | 4 (0.6) |
| Others | 17 (4.7) | 20 (7.8) | 37 (5.9) |
| Total N (%) | 365 (100.0) | 257 (100.0) | 622 (100.0) |
|  |  |  |  |
| **Cause of death*** |  |  |  |
| Cong.Malform./Sys.dis | 5 (29.4) | 4 (26.7) | 9 (28.1) |
| ARI+CHD | 2 (11.8) | 3 (20.0) | 5 (15.6) |
| Sudden Infant Death (SID)****** | 4 (23.5) | 1 (6.7) | 5 (15.6) |
| Pneumonia/ARI | 3 (17.6) | 1 (6.7) | 4 (12.5) |
| Acute Meningitis | 2 (11.8) | 1 (6.7) | 3 (9.4) |
| Diarrohea | 0 (0.0) | 2 (13.3) | 2 (6.3) |
| Sepsis | 1 (5.9) | 0 (0.0) | 1 (3.1) |
| Others | 1 (5.9) | 2 (13.3) | 3 (9.4) |
| Total N (%) | 17 (100.0) | 15 (100.0) | 32 (100.0) |
| Notes: See Table 2; *The specific causes of death includes: congenital heart diseases (n=6; single ventricle, ventricular septum defect with severe pulmonary arterial hypertension, atrio-ventricular septum defect, dilated cardiomyopathy); other systemic malformations (n=5; congenital biliary atresia, pelviureteric junction obstruction, meningomyelocele with hydrocephalus, spinal muscular atrophy) and chromosomal disorders (n=4; mucopolysacharidosis, dysmorphism, thanatophoric dysplasia, congenital adrenal hyperplasia) and infections (n=12); and ******sudden infant death (n=5) includes one each in 0-6, 7-13, 14-20 and 21-27days of risk-periods, respectively | | | |
