# Supplementary material for: A Prospective Cohort Study on the Safety of Infant Pentavalent (DTwP-HBV-Hib) and Oral Polio Vaccines in Two South Indian Districts
Source: Pediatr Infect Dis J. 2020 Apr 14;39(5):389–96. doi: 10.1097/INF.0000000000002594 (PMC7170438; doi:10.1097/INF.0000000000002594)
Supplement: Supplementary file 3 [file inf-39-389-s003.docx]

**Supplemental Digital Content 3.** All-cause hospitalization (within four weeks) incidence rates and incidence rate-ratios (unadjusted & adjusted) after pentavalent and oral polio vaccines: Combined for both districts, Kollam (Kerala) and Coimbatore (Tamil Nadu), India.

| Vaccine dose/  Risk period  (in days)* | Total number of | | Incidence-rate  (IR, with exact 95% CI) | Incidence rate-ratios (IRRs, with 95% CI) | | | |
| --- | --- | --- | --- | --- | --- | --- | --- |
|  | Events | Person-time (in days) |  |  |  |  |  |
|  |  |  |  | Unadjusted | p-value | Adjusted† | p-value |
| **Dose-1** |  |  |  |  |  |  |  |
| 0-6 days | 35 | 214702 | 16.3 (11.4–22.7) | 0.9 (0.6–1.5) | 0.717 | 1.2 (0.7–1.9) | 0.492 |
| 7-13 days | 22 | 214740 | 10.2 (6.4–15.5) | 0.6 (0.3–1.0) | 0.040 | 0.7 (0.4–1.2) | 0.154 |
| 14-20 days | 25 | 214694 | 11.6 (7.5–17.2) | 0.7 (0.4–1.1) | 0.102 | 0.7 (0.4–1.2) | 0.188 |
| 21-27 days^‡^ | 38 | 214158 | 17.7 (12.6–24.4) | Reference |  | Reference |  |
| **Dose-2** |  |  |  |  |  |  |  |
| 0-6 days | 29 | 213472 | 13.6 (9.1–19.5) | 0.7 (0.4–1.1) | 0.148 | 0.9 (0.5–1.4) | 0.522 |
| 7-13 days | 33 | 213400 | 15.5 (10.6–21.7) | 0.8 (0.5–1.3) | 0.344 | 0.9 (0.6–1.5) | 0.691 |
| 14-20 days | 34 | 213306 | 15.9 (11.0–22.3) | 0.8 (0.5–1.3) | 0.411 | 0.9 (0.6–1.4) | 0.579 |
| 21-27 days^‡^ | 41 | 212523 | 19.3 (13.8–26.2) | Reference |  | Reference |  |
| **Dose-3** |  |  |  |  |  |  |  |
| 0-6 days | 14 | 211294 | 6.6 (3.6–11.1) | 0.4 (0.2–0.8) | 0.007 | 0.5 (0.3–1.0) | 0.035 |
| 7-13 days | 38 | 210847 | 18.0 (12.8–24.7) | 1.1 (0.7–1.8) | 0.576 | 1.3 (0.8–2.1) | 0.288 |
| 14-20 days | 23 | 210418 | 10.9 (6.9–16.4) | 0.7 (0.4–1.2) | 0.177 | 0.7 (0.4–1.3) | 0.255 |
| 21-27 days^‡^ | 33 | 209168 | 15.8 (10.9–22.2) | Reference |  | Reference |  |
| **All 3-doses** |  |  |  |  |  |  |  |
| 0-6 days | 78 | 639468 | 12.2 (9.6–15.2) | 0.7 (0.5–0.9) | 0.013 | 0.7 (0.6–1.0) | 0.047 |
| 7-13 days | 93 | 638987 | 14.6 (11.8–17.8) | 0.8 (0.6–1.1) | 0.174 | 0.9 (0.7–1.1) | 0.301 |
| 14-20 days | 82 | 638418 | 12.8 (10.2–15.9) | 0.7 (0.5–1.0) | 0.030 | 0.7 (0.6–1.0) | 0.043 |
| 21-27 days^‡^ | 112 | 635849 | 17.6 (14.5–21.2) | Reference |  | Reference |  |
| **Sensitivity analysis** |  |  |  |  |  |  |  |
| 0-6 days of Dose- 2 | 29 | 213472 | 13.6 (9.1–19.5) | 0.8 (0.5–1.2) | 0.279 | 0.6 (0.4–1.0) | 0.072 |
| 21-27 days of Dose-1^‡^ | 38 | 214158 | 17.7 (12.6–24.4) | Reference |  | Reference |  |
| 0-6 days of Dose-3 | 14 | 211294 | 6.6 (3.6–11.1) | 0.3 (0.2–0.6) | 0.001 | 0.3 (0.2–0.6) | 0.001 |
| 21-27 days of Dose-2^‡^ | 41 | 212523 | 19.3 (13.8–26.2) | Reference |  | Reference |  |
| **See* as in Table 2. | | | | | | | |
