# Supplementary material for: A Prospective Cohort Study on the Safety of Infant Pentavalent (DTwP-HBV-Hib) and Oral Polio Vaccines in Two South Indian Districts
Source: Pediatr Infect Dis J. 2020 Apr 14;39(5):389–96. doi: 10.1097/INF.0000000000002594 (PMC7170438; doi:10.1097/INF.0000000000002594)
Supplement: Supplementary file 4 [file inf-39-389-s004.docx]

**Supplemental Digital Content 4.** All-cause appropriate hospitalization (within four weeks) incidence rates and incidence rate-ratios (unadjusted & adjusted) after pentavalent and oral polio vaccines: Combined for both districts, Kollam (Kerala) and Coimbatore (Tamil Nadu), India.

| Vaccine dose/  Risk period  (in days)* | Total number of | | Incidence-rate  (IR, with exact 95% CI) | Incidence rate-ratios (IRRs, with 95% CI) | | | |
| --- | --- | --- | --- | --- | --- | --- | --- |
|  | Events | Person-time(in days) |  |  |  |  |  |
|  |  |  |  | Unadjusted | p-value | Adjusted† | p-value |
| **Dose-1** |  |  |  |  |  |  |  |
| 0-6 days | 28 | 214727 | 13.0 (8.7–18.9) | 0.9 (0.5–1.5) | 0.689 | 1.2 (0.7–2.0) | 0.578 |
| 7-13 days | 19 | 214748 | 8.9 (5.3–13.8) | 0.6 (0.3–1.1) | 0.091 | 0.7 (0.4–1.3) | 0.268 |
| 14-20 days | 18 | 214716 | 8.4 (5.0–13.3) | 0.6 (0.3–1.0) | 0.065 | 0.6 (0.4–1.1) | 0.119 |
| 21-27 days^‡^ | 31 | 214172 | 14.5 (9.8–20.6) | Reference |  | Reference |  |
| **Dose-2** |  |  |  |  |  |  |  |
| 0-6 days | 24 | 213480 | 11.2 (7.2–16.7) | 0.8 (0.5–1.4) | 0.483 | 1.0 (0.6–1.8) | 0.939 |
| 7-13 days | 24 | 213420 | 11.3 (7.2–16.7) | 0.8 (0.5–1.4) | 0.484 | 1.0 (0.6–1.6) | 0.864 |
| 14-20 days | 29 | 213318 | 13.6 (9.1–19.5) | 1.0 (0.6–1.7) | 0.989 | 1.1 (0.6–1.8) | 0.795 |
| 21-27 days^‡^ | 29 | 212565 | 13.6 (9.1–19.6) | Reference |  | Reference |  |
| **Dose-3** |  |  |  |  |  |  |  |
| 0-6 days | 11 | 211307 | 5.2 (2.6–9.3) | 0.4 (0.2–0.8) | 0.016 | 0.5 (0.3–1.1) | 0.069 |
| 7-13 days | 30 | 210877 | 14.2 (9.6–20.3) | 1.1 (0.7–1.9) | 0.614 | 1.3 (0.8–2.2) | 0.313 |
| 14-20 days | 19 | 210423 | 9.0 (5.4–14.1) | 0.7 (0.4–1.3) | 0.290 | 0.8 (0.4–1.4) | 0.398 |
| 21-27 days^‡^ | 26 | 209182 | 12.4 (8.1–18.2) | Reference |  | Reference |  |
| **All 3-doses** |  |  |  |  |  |  |  |
| 0-6 days | 63 | 639514 | 9.9 (7.6–12.6) | 0.7 (0.5–1.0) | 0.056 | 0.8 (0.6–1.1) | 0.167 |
| 7-13 days | 73 | 639045 | 11.4 (9.0–14.4) | 0.8 (0.6–1.2) | 0.289 | 0.9 (0.7–1.2) | 0.474 |
| 14-20 days | 66 | 638457 | 10.3 (8.0–13.2) | 0.8 (0.6–1.1) | 0.101 | 0.8 (0.6–1.1) | 0.138 |
| 21-27 days^‡^ | 86 | 635919 | 13.5 (10.8–16.7) | Reference |  | Reference |  |
| **Sensitivity analysis** |  |  |  |  |  |  |  |
| 0-6 days of Dose- 2 | 24 | 213480 | 11.2 (7.2–16.7) | 0.8 (0.5–1.3) | 0.353 | 0.7 (0.4–1.1) | 0.123 |
| 21-27 days of Dose-1^‡^ | 31 | 214172 | 14.5 (9.8–20.6) | Reference |  | Reference |  |
| 0-6 days of Dose-3 | 11 | 211307 | 5.2 (2.6–9.3) | 0.4 (0.2–0.8) | 0.007 | 0.3 (0.2–0.7) | 0.004 |
| 21-27 days of Dose-2^‡^ | 29 | 212565 | 13.6 (9.1–19.6) | Reference |  | Reference |  |
| **See* as in Table 2; and appropriate hospitalizations (Total) as per expert opinion and identified using appropriateness (PAEP-India) criteria. | | | | | | | |
